# Self-Assembling Peptide Detergents Stabilize Isolated Photosystem Ion a Dry Surface for an Extended Time

**DOI:** 10.1371/journal.pbio.0030230

**Published:** 2005-06-21

**Authors:** Patrick Kiley, Xiaojun Zhao, Michael Vaughn, Marc A Baldo, Barry D Bruce, Shuguang Zhang

**Affiliations:** **1**Center for Biomedical Engineering NE47–379, Massachusetts Institute of Technology, Cambridge, Massachusetts, United States of America,; **2**Department of Electrical Engineering and Computer Science, Massachusetts Institute of Technology, Cambridge, Massachusetts, United States of America,; **3**Center for Environmental Biotechnology and Department of Biochemistry, Cellular and Molecular Biology, University of Tennessee, Knoxville, Tennessee, United States of America,; **4**Center for Bits and Atoms, Massachusetts Institute of Technology, Cambridge, Massachusetts, United States of America; Brandeis UniversityUnited States of America

## Abstract

We used a class of designed peptide detergents to stabilize photosystem I (PS-I) upon extended drying under N_2_ on a gold-coated-Ni-NTA glass surface. PS-I is a chlorophyll-containing membrane protein complex that is the primary reducer of ferredoxin and the electron acceptor of plastocyanin. We isolated the complex from the thylakoids of spinach chloroplasts using a chemical detergent. The chlorophyll molecules associated with the PS-I complex provide an intrinsic steady-state emission spectrum between 650 and 800 nm at −196.15 °C that reflects the organization of the pigment-protein interactions. In the absence of detergents, a large blue shift of the fluorescence maxima from approximately 735 nm to approximately 685 nm indicates a disruption in light-harvesting subunit organization, thus revealing chlorophyll−protein interactions. The commonly used membrane protein-stabilizing detergents, N-dodecyl-β-D-maltoside and N-octyl-β-D-glucoside, only partially stabilized the approximately 735-nm complex with approximately 685-nm spectroscopic shift. However, prior to drying, addition of the peptide detergent acetyl- AAAAAAK at increasing concentration significantly stabilized the PS-I complex. Moreover, in the presence of acetyl- AAAAAAK, the PS-I complex is stable in a dried form at room temperature for at least 3 wk. Another peptide detergent, acetyl-VVVVVVD, also stabilized the complex but to a lesser extent. These observations suggest that the peptide detergents may effectively stabilize membrane proteins in the solid-state. These designed peptide detergents may facilitate the study of diverse types of membrane proteins.

## Abstract 2

A designer peptide detergent may facilitate the study of diverse types of membrane proteins.

## Introduction

The bioinformatics and computational analyses of a large number of completely sequenced genomes suggest that over 30% of all genes encode membrane proteins [1–4]. These predicted membrane proteins, identified to contain at least one *trans*-membrane domain, participate in a plethora of vital cellular activities, including cell signaling, cell–cell interactions, cell adhesion, cell migration and movement, cytoskeleton organization, protein trafficking, viral fusion, secretory and synaptic activities, ion and metabolite transport, and respiratory and photosynthetic electron transport. However, many detailed structures of diverse membrane proteins remain elusive. Thus the discovery and design of new detergents are acutely needed to facilitate membrane protein crystallization and structural studies [4,5].

Photosystem I (PS-I) is a membrane protein complex that is associated with electron transport and exists as a large, multisubunit complex with dozens of transmembrane-spanning domains [6,7]. The composition and organization of PS-I complexes have presented a great challenge in the development of experimental conditions for efficient isolation, while maintaining the native organization of the proteins, associated cofactors, prosthetic groups, and their biological functions. The largest membrane complex isolated and crystallized to date is the plant photosynthetic reaction center, PS-I [7]. This plant PS-I complex has 16 subunits, containing 45 transmembrane domains and 167 chlorophyll molecules. Because of its antennae size, the production of a low potential reductant, and its nanoscale structure, PS-I has recently been shown to be an attractive choice for integration into solid-state biophotovoltaic devices [8].

In order to fabricate protein-based devices, it is crucial to immobilize and stabilize the selected proteins and enzymes for an extended time. Although some progress has been made [9–11], little has been reported on the successful immobilization of membrane proteins on surfaces [12–15]. Several investigators have reported that bacteriorhodopsin can be immobilized by entrapment in dried xerogel glass and retains partial activity for extended periods [16,17].

The difficulties associated with solubilization and stabilization of membrane proteins are largely due to exposure of their hydrophobic domains, which are normally buried within the lipid bilayer. These hydrophobic transmembrane segments render the protein particularly difficult to stabilize after removal from the membrane. In particular, they are extremely susceptible to aggregation in aqueous media. In previous decades, a wealth of chemical detergents was synthesized for the study of membrane proteins (http://www.anatrace.com/). In some cases these detergents have partly facilitated selective solubilization of membrane proteins from their native environments. Recent evidence suggests, for example, that detergents with short alkyl chains are more effective at solubilization than those with long alkyl chains that often lead to protein denaturation [18,19]. These new chemical detergents have advanced the study of membrane proteins.

Some efforts have been made to design helical peptide- and protein-based detergents [20,21]. Stroud and colleagues designed the first α-helix peptide detergent, called a *peptitergent,* which has a hydrophilic face and a hydrophobic face [20]. The peptide formed a four-helical bundle in solution when not interacting with membrane proteins. They showed that this peptitergent can solubilize 85% bacteriorhodopsin and approximately 60% rhodopsin for over 2 d [20]. Privé and colleagues also designed a monomeric α-helix coupled with two alkyl chains at both N- and C-termini. This hybrid molecule has two faces, one hydrophilic face from the charged residues on one side of the helix that interacts with water and a hydrophobic face on the other that interacts with transmembrane domains of membrane proteins. They showed that this type of hybrid peptide–alkyl detergent solubilized and stabilized several membrane proteins [21]. Several other designed helical peptide detergents have been shown to solubilize membrane proteins as well [22–24]. However, these peptides require efficient folding, are not easily accessible, are expensive to produce, and are difficult to make for large-scale productions and widespread studies.

We have designed and studied a new class of short self-assembling peptide detergents, which are six to eight residues long, for their ability to maintain solubility of membrane proteins. The behavior of these peptide detergents in the absence of proteins has been previously described [25–28]. In the absence of proteins, these detergent peptides form stable nanotubes, nanovesicles, and micelles when they are above their critical aggregation concentration (CAC). The behavior of these peptide detergents is similar to that of lipids and other chemical detergents. It is plausible that the protein–peptide detergent interactions may have enhanced stabilizing capability over short-chain alkane compounds since these peptide detergents share similar chemical properties. This increased stability has recently proven useful in the incorporation of spinach PS-I into a solid-state photovoltaic device [8].

We here report the stabilization of spinach PS-I complex following chemical detergent isolation. We studied the ability of these peptide detergents to interact with and stabilize isolated spinach PS-I complexes that have been dried under N_2_ on glass slides. The complex stability and organization of the chlorophyll was monitored via the −196.15 ^°^C steady-state emission spectrum of PS-I. We showed that the PS-I complex alone became unstable during the drying process or in the presence of chemical detergents. However, we further demonstrate that one of the designed peptide detergents, acetyl- AAAAAAK (A_6_K), is not only able to preserve the original spectral properties of the PS-I complex, but also stabilizes PS-I in the dry form for a period of at least 3 wk. In light of the recently reported pea PS-I crystal structure [7], we also discuss how the chlorophyll pigment–protein complexes may be influenced during dehydration.

## Results

### Isolation of Spinach PS-I

To evaluate the effect of the peptide detergents for stabilizing membrane proteins, we isolated the chlorophyll pigment protein from spinach chloroplast thylakoids. PS-I was isolated from spinach using a modified procedure of Mullet and Arntzen [29]. The typical results of the continuous sucrose gradient used for isolation are shown in Figure 1. The large green band on the bottom 2.2-M sucrose cushion is collected using a needle syringe (Figure 1A). The additional chlorophyll at the top of the gradient is from LHCPII and PS-II. This preparation has a typical chlorophyll/P700 ratio of approximately 200 and a chlorophyll a/b ratio of 4.75 (data not shown). The SDS-PAGE gel analysis (Figure 1B) of the preparation showed that the sample is in agreement with literature. The chlorophyll is associated with the PsaA and PsaB polypeptides that run as a diffuse band at greater than 65 kDa and the set of LHCI polypeptides shown with molecular weight in the range of 20–26 kDa. The minor band at approximately 30 kDa is due to the LHCPII antennae from PS-II.

**Figure 1:**
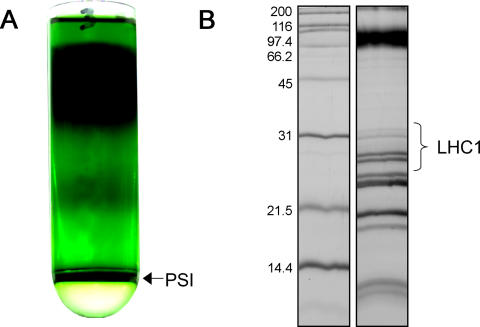
Isolation of PS-I by Detergent Solubilization and Density-Gradient Centrifugation (A) The final centrifugation step of the isolation protocol, the linear 0.1–1M sucrose gradients contain 0.02% Triton X-100. The detergent-solubilized (at 0.8% v/v Triton X-100) PS-I particles selectively aggregate upon entering the low Triton X-100 concentration of the gradient and sediment quickly relative to the other thylakoid proteins that remain at the top of the gradient. (B) SDS-PAGE 18% slab gel lane containing the PS-I harvested from the 1M/2M interface as seen in (A). The region containing the LHCI is indicated.

### PS-I Stability in Various Conditions

For PS-I complex dried in the absence of peptide detergent, a significant change in the fluorescence spectrum was observed. This change consisted of a decrease in the overall intensity of the far-red fluorescing chlorophyll in the approximately 735-nm region and an associated blueshift of this peak. In addition, the fluorescence peak at approximately 680 nm demonstrated an overall increase in intensity after the drying process. In some cases, a blueshift of this peak was also observed. The emission spectra of dried and solution state samples in the present chemical detergents are shown in Figure 2.

**Figure 2:**
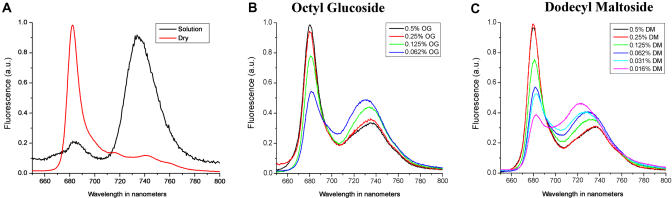
The −196.15 °C Steady-State Emission Spectra of PS-I (A) The solution spectrum of PS-I shows characteristic far-red fluorescence at the approximately 735-nm region, while dried PS-I shows a peak at approximately 683 nm. (B) Emission spectra of samples containing DM retained partially their far-red fluorescence, but still showed a significant increase in fluorescence at approximately 683 nm. Additionally, the far-red fluorescence demonstrated a significant blueshift. (C) Comparable changes are evident in the spectra of samples containing OG as seen in DM (B).

We used four widely known membrane protein-stabilizing detergents, Triton X-100, N, N-dimethyldodecylamine N-oxide, N-dodecyl-β-D-maltoside (DM), and N-octyl-β-D-glucoside (OG) for the PS-I complex. Triton X-100 and N, N-dimethyldodecylamine N-oxide did not demonstrate a stabilizing effect based on the emission spectrum of PS-I, but in most cases caused increased degradation of the complex (data not shown). On the other hand, OG and DM showed modest stabilizing effect at low concentrations. The spectra for OG and DM are shown in Figure 2. At low concentration, both OG and DM resulted in a blueshift from the approximately 735-nm region, but at higher concentration, both reduced the fluorescent emission at approximately the 735-nm region, with an increasing emission at 680 nm, implying they reduced PS-I stability. Thus, these results suggest that although OG and DM are good detergents for solubilization and stabilization of some membrane proteins, they are not ideal stabilizing detergents for PS-I under our conditions.

### Stabilizing the PS-I Complex Using Peptide Detergents

We carried out systematic experiments to determine if the peptide detergents A_6_K (molecular weight = 615 Da, CAC = 1.6 mM in water, approximately 0.23 mM in phosphate-buffered saline [PBS]) and acetyl-VVVVVVD (V_6_D) (molecular weight = 783 Da, CAC = 0.7 mM in water, approximately 0.1 mM in PBS) could stabilize PS-I complex during the drying process. Samples were prepared by addition of A_6_K or V_6_D at concentrations of 0.016%, 0.031%, 0.062%, 0.125%, 0.25%, and 0.5% (A_6_K: 0.5% = 8 mM, or 34X CAC in PBS; V_6_D: 0.5% = 6.4 mM, or 64X CAC in PBS). The results are shown in Figure 3. Under these conditions at lower concentrations of A_6_K or V_6_D, PS-I not only showed blueshift but also showed a reduced fluorescent emission after drying. On the other hand, at higher concentration, the PS-I complex appeared to be stabilized during and after drying. In particular, samples containing 0.5% A_6_K demonstrated essentially negligible change in their emission spectra in the approximately 735-nm region. V_6_D was less effective at stabilizing PS-I as shown in Figure 3B, even at the highest concentration of 0.5%.

**Figure 3:**
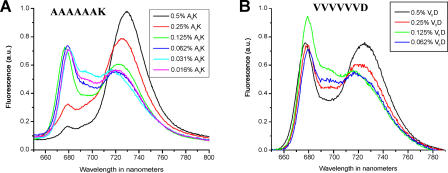
Emission Spectra of the Dried PS-I Complex Containing Various Concentrations of the Peptide Detergents A_6_K and V_6_D (A) A_6_K showed significantly enhanced ability to stabilize PS-I at a concentration of 0.5% (w/v) and showed reduced ability to stabilize PS-I complex at decreasing concentration. (B) V_6_D showed lesser ability to stabilize PS-I complex at the same concentration.

### The Peptide Detergent A_6_K Stabilizes the PS-I Complex for an Extended Time

We asked how long the peptide detergent A_6_K could stabilize the PS-I complex on a dry surface. The stability of dried PS-I with 0.5% A_6_K (34X CAC in PBS) on a glass slide was measured at days 1, 7, 14, and 21. The results are shown in Figure 4. A noticeable blueshift in the approximately 735-nm region coupled with a decrease in the intensity is apparent. At the same time, a slight increase of intensity in the approximately 680-nm region is visible. Despite this shift, the overall change in shape in the spectrum is negligible in contrast to other drastic changes observed with other detergents. Our observations suggest that the A_6_K peptide detergent is able to stabilize the PS-I complex under the conditions used.

**Figure 4:**
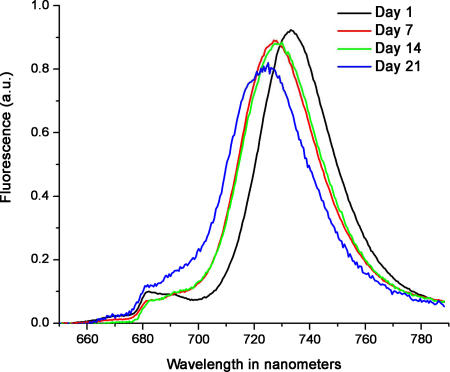
Extended Time of PS-I Stability on Dried Surface Fluorescence spectrum of a PS-I film containing 0.5% A_6_K was monitored for 3 wk. Negligible increase in fluorescence was observed at approximately 683 nm. A blueshift from approximately 735 nm to approximately 727 nm fluorescence was observed. Thus, it suggests that the PS-I complex retained most of its structural integrity under conditions examined. Such shifts have been noted under other conditions.

This observation is consistent with a separate experiment of another membrane protein, rhodopsin, using other peptide detergents. In these experiments, rhodopsin was stabilized for an extended time when high concentrations of peptide detergents were used (X. Zhao et al., unpublished data).

## Discussion

### PS-I Spectroscopy Features

Study of the low-temperature chlorophyll fluorescence in the approximately 735-nm region was originated over 25 y ago [30]. It was initially presumed to be due to a chlorophyll species C-705 that was only observable at low temperatures because of its ability to undergo rapid energy transfer to P700 at elevated temperatures. However, a careful analysis of PS-I, with its full antennae complement, has demonstrated the presence of at least three far-red chlorophyll spectral forms with emission maxima of 720 nm, 730 nm, and 742 nm [31]. Recently, the longest wavelength fluorescence band has been observed even at room temperature using selective excitation, and has been shown to be associated with the lowest-energy spectral form of LHCI antenna complex [32].

The ability to monitor the reversible dissociation of pigment–protein complexes and the consequent cessation of intercomplex energy transfer by the relative intensities of −196.15 °C fluorescence at 685 nm and 735 nm has long been realized [33]. Monitoring of the F685/F735 chlorophyll fluorescence ratio has been used to study many in vivo and in vitro biological processes, including (1) the effect of leaf temperature [34], (2) seasonally induced chlorophyll breakdown in trees [35], (3) herbicide phytotoxicity in algae [36], (4) high-light-induced photoinhibition [37], (5) iron deficiency in algae [38], and (6) PS-I stability and disassembly [39].

Studies have also been carried out on the −196.15 °C emission spectrum of the PS-I core. Based on these studies, in which there is significant fluorescence at approximately 715 nm, it is presumable that damage could be done to the PS-I during drying as well. It is interesting to note that similar results have been reported from adverse interactions with detergent [31]. In these studies, it is argued that the fluorescence change observed is the result of uncoupled chlorophyll molecules.

### Analyses of PS-I Structure and Function

Much progress has been made in the study of PS-I, mostly through the combination of membrane fractionation studies, various types of quantitative spectroscopy, protein crystallography and structural analyses. The chlorophyll pigment proteins responsible for these various spectrally distinct species are finally being resolved [6,7]. The −196.15 °C steady state emission spectrum of plant PS-I has been attributed to the fluorescence of the LHCI proteins [40] associated with the core complex, which themselves demonstrate an extremely redshifted chlorophyll fluorescence [41–43].

The spectral changes presented here could therefore be the result of changes in the LHCI peptides, or in their association with the PS-I core complex. Several experiments on LHCI peptides have given similar results to those presented here. For example, it was shown that heterodimerization was an essential feature of LHC-730 [44]. The fluorescence at 730 nm occurred only for the dimeric state. Furthermore, site-directed mutagenesis studies of *lhca*1–4 genes showed that the approximately 730-nm emission could be abolished with a commensurate increase in the fluorescence at approximately 680 nm [45] by altering the specific interaction between protein and chlorophyll. It has been suggested that the LHC-680 peptides show an emission maximum at approximately 680 nm only in the monomeric form [46,47]. These studies further indicate that such spectral changes are the result of a disruption of how the LHCI peptides and their associated chlorophyll are “connected” to the PS-I core complex.

The current understanding for chlorophyll distribution in native PS-I including the antennae LHCI is that the core complex has approximately 110 chlorophyll molecules associated with PsaA/B. The peripheral antennae contain approximately 84 chlorophyll molecules composed of four distinct subunits, LhcA1–4, that exist in the form of heterodimers. These heterodimers are isolated as two fractions: LHCI-A (composed of LhcA1 and LhcA4) and LHCI-B (composed of LhcA2 and LhcA3). From analysis of chlorophyll content of isolated complexes and reconstituted complexes, each LHCI monomer subunit appears to bind approximately 10 chlorophyll molecules. This would then suggest that the native complex with approximately 195 chlorophyll molecules must have four heterodimers associated with each core complex. Undoubtedly, the precise number of antenna LHCI subunits will vary with plant species, plant age, and environmental conditions. In fact, analysis of the recently solved crystal structure of pea PS-I, which contains only 167 chlorophyll molecules, reveals that there are only two heterodimers. These four LHCI subunits that are asymmetrically positioned on one side of the PS-I core complex form an “LHCI belt” (Figure 5).

**Figure 5:**
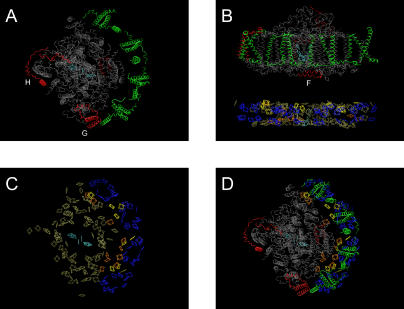
Crystal Structure of PS-I as Determined by Ben-Shem et al. [7,53], Visualized in VMD [52], and Rendered with POV-Ray 3.6 The color scheme is adapted from Ben-Shem et al. [7,53] and is interpreted as follows. Portions shown in gray are common subunits found in both PS-I reaction centers in cyanobacteria and higher plants, while the red subunits are unique to higher plants. The two LHCI heterodimers are shown in green. The two different orientations of selected PS-I complex are shown top-down (A) and in a side view (B). In (C) and (D) the chlorophyll molecules are colored by a putative function: the P700 core antennae are tan, the special pair (P700) and voyeur chlorophyll molecules are cyan, the linker chlorophyll between the LHCI heterodimers is in yellow, the antennae chlorophyll molecules complexed within an LHCI subunit are in blue, and the “gap” chlorophyll molecules are shown in orange. (A), (C), and (D) are views looking down on the stromal hump highlighting protein, chlorophyll, and composite, respectively. (B) faces the LHC belt from inside the plane of the thylakoid membrane; only protein is shown in the upper portion and only chlorophyll below.

The large Mg^2+^–Mg^2+^ distances between the core complex chlorophyll molecules and the primary LHCI chlorophyll molecules would suggest that energy transfer between the LHCI belt and core complex is directed through specialized “gap” chlorophyll molecules at the junction of LhcA1 and subunit G (Figure 5A). It is believed that this region in the native protein may coordinate several LHCI subunits. Apparently, the optimization of energy transfer between LHCI complexes and the core complex has been facilitated through the evolution of a select number of strategically placed chlorophyll-binding sites that permit coordination of “gap” chlorophyll molecules [7].

These “gap” chlorophyll molecules in orange are shown in Figure 5C. Interestingly, these “gap” chlorophyll molecules are positioned in a relatively open part of the structure, which may be highly vulnerable to the conformational changes of the complex following either dehydration or excessive detergent solubilization. The stabilizing properties of the detergent peptides may stem from their appropriate dimensions, allowing insertion into the complex so that they favorably stabilize these “gap” chlorophyll molecules, thus maintaining their low-temperature fluorescence properties. In this work, the PS-I complex that was dried with 0.5% A_6_K demonstrated minimal changes in its emission spectra, suggesting that there has been minimal change in the chlorophyll associated with PS-I. The most noticeable difference was a slight blueshift in the emission from approximately 735 nm to approximately 727 nm, associated with the far-red fluorescing chlorophyll, and a negligible increase in the approximately 680-nm region fluorescence. This change has also been reported before, and it is considered the result of a slight decoupling of the LHCI peptides from the PS-I complex. We therefore propose that the stabilizing effect of A_6_K and V_6_D on PS-I can be determined by the ratio of the fluorescence at approximately 730 nm to the fluorescence at approximately 680 nm.

### Designed Peptide Detergents for the Study of Membrane Proteins

PS-I is one of the first examples that clearly demonstrate how designed peptide detergents can stabilize membrane proteins. In collaboration with others, we have also stabilized bovine rhodopsin with the peptide detergents A_6_D and V_6_D for an extended time at elevated temperatures (X. Zhao et al., unpublished data). In collaboration with another investigator, using the peptide detergent V_6_D, a crystal of the integral membrane protein glycerol 3-phosphate dehydrogenase with its cofactor was obtained and was diffracted to approximately 7 Å using an in-house X-ray diffraction instrument (J. Yeh et al., unpublished data).

It is plausible that peptide detergents, similar to other detergents, may directly interact with the hydrophobic domains of membrane proteins. It is likely that numerous peptide detergent molecules, approximately 2 nm in size, like lipids, can effectively surround the hydrophobic *trans*-membrane domains of membrane proteins, thus sequestering them from directly interacting with water molecules and preventing them from undergoing self-aggregation. In the PS-I complex, the peptide detergents not only sequestered the protein and filled the “gaps” but also maintained its overall structural integrity, thus retaining its biological function [8].

The applications of designed peptide detergents are still in an early stage of development. The peptide detergents presented here represent a new class of bioengineered material because they can be designed at the molecular level, using a combinatorial approach characteristic of peptide chemistry. However, we have yet to introduce the variability inherent in amino acids. For example, so far, we have concentrated on peptides containing one kind of hydrophobic tail. It is likely that as we explore combinations of short and long tails, as well as different head groups, the molecules will become more specialized, and we may find specific sequences that work best for stabilizing specific membrane proteins. Equally useful is the exploration of mixing several different detergents together as a cocktail. In essence, this could let the protein select the most appropriate mixture for stabilization and crystallization.

## Materials and Methods

### PS-I isolation and electrophoresis

PS-I was obtained from spinach leaves by homogenization in 400 mM sorbitol and 50 mM tricine-KOH (pH 7.8). The resulting homogenate was then passed through Miracloth (approximately 30 μm; CalBiochem, San Diego, California, United States), supported by a double layer of cheesecloth, and centrifuged at 1,000 × g for 5 min. The pellet, consisting mostly of chloroplasts and broken plastids, was resuspended and forced through five passes in a Dounce homogenizer in 50 mM sorbitol, 5 mM EDTA-NaOH, and 50 mM tricine-KOH (pH 7.8), which lysed any unbroken plastids. It was centrifuged again at 10,000 × g for 5 min; this step was repeated once. The resulting pellet was highly enriched in thylakoids, which were completely nonappressed after treatment with the chelator; this pellet was resuspended in a small volume of water, and the chlorophyll content was determined. The thylakoid suspension was then brought to a final concentration of 0.8 mg/ml chlorophyll and 0.8% (w/v) Anatrace (http://www.anatrace.com/) Anapoe Triton X-100. Gentle stirring began immediately at room temperature for 30 min. Following the incubation, the solution was spun at 42,000 × g for 30 min, and the pellet was discarded. Approximately 8 ml of the supernatant was loaded onto approximately 25 ml linear 0.1–1 M sucrose gradients containing 0.02% Triton X-100 with 2 ml of 2M sucrose (no Triton) as a cushion and spun at 100,000 × g in a Beckman SW-28 rotor overnight (15 h). The dark green band at the 1M–2M interface was harvested with a long needle and passed through a Dounce homogenizer, pooled, and frozen at −80 °C. The protocol was adapted from Mullet et al. [48].

### Peptide synthesis and preparation

Peptides were obtained from the MIT Biopolymers Laboratory and Synpep Corporation (www.synpep.com). The peptide detergent stock solutions were made by dissolving these peptides at a concentration of 1% (w/v) in 50 mM Tris (pH 7.8); 25 mM NaCl; 0.02% Triton X-100, and then sonicating in a water bath sonicator for 20 min. Stock solutions of peptides were stored at room temperature for the duration of the experiment.

### PS-I treatment with detergents and peptides

This was carried out by diluting 25 μl of PS-I with 25 μl of serial dilutions of the peptide stock solutions in buffer. This resulted in a chlorophyll concentration of 50 μg/ml in all samples and peptide concentrations of 0.5%, 0.25%, 0.125%, 0.063%, 0.032%, and 0.016%. For most experiments, the peptide detergent was used at final 0.5% concentration.

### PS-I dehydration

All samples were flash frozen in liquid nitrogen until use. These samples were then thawed and dried on 1-cm^2^ glass slides for at least 15 h under N_2_.

### Low-temperature fluorescence measurements

Their steady-state emission spectra at −196.15 °C [31,49–51] were then determined to investigate the stability of the PS-I in each sample. Each slide was cooled under liquid nitrogen in a cryostat, and fluorescence was excited optically using a 408-nm laser. Steady-state emission spectra were recorded using a CCD spectrometer with an optical fiber input oriented 90° from the laser and a slit width of 20 μm. The fluorescence intensity of each sample was normalized to the integral over the wavelength shown.

### Modeling of pea PS-I

The structure of pea PS-I was determined at 4.4 Å [7]. We highlighted in Figure 5 the components of this structure that may add to our understanding of the fluorescence phenomena reported in this work. Manipulations of the molecular coordinates were made with VMD 1.8.2 [52], and the images were rendered in POV-Ray 3.6 (http://www.povray.org /download/).

## Supporting Information

### Accession Number

The Protein Data Bank (http://www.rcsb.org/pdb/) accession number for the crystal structure of pea PS-I is 1QZV.

## Abbreviations

A6K: acetyl- AAAAAAK
CAC: critical aggregation concentration
DM: N-dodecyl-β-D-maltoside
OG: N-octyl-β-D-glucoside
PBS: phosphate-buffered saline
PS-I: photosystem I
V6D: acetyl-VVVVVVD
